# Exosome-mediated communication in the tumor microenvironment contributes to hepatocellular carcinoma development and progression

**DOI:** 10.1186/s13045-019-0739-0

**Published:** 2019-05-29

**Authors:** Qin Wu, Lingyun Zhou, Duoduo Lv, Xia Zhu, Hong Tang

**Affiliations:** 1 Center of Infectious Diseases, West China Hospital, Sichuan University, Chengdu, China; 2 Division of Infectious Diseases, State Key Laboratory of Biotherapy and Center of Infectious Diseases, West China Hospital, Sichuan University, Chengdu, China

**Keywords:** Exosomes, Tumor microenvironment, Hepatocellular carcinoma, Immune regulation, Therapy

## Abstract

The tumor microenvironment (TME) is an essential intrinsic portion of hepatocellular carcinoma (HCC) for the regulation of its origination, development, invasion, and metastasis. As emerging components of the tumor-host interaction, exosomes are increasingly recognized as professional carriers of information in TME and as pivotal molecular entities involved in tumorigenic microenvironment setup. However, much remains unknown about the role of the exosome communication system within TME in the development and progression of HCC. In this review, we focus on the roles and probable mechanisms of TME in HCC and show the exosome-based immune regulation in TME to promote HCC. Multiple processes are involved in HCC, including tumor survival, growth, angiogenesis, invasion, and metastasis. We also discuss the specific roles of exosomes in HCC processes by molding hospitable TME for HCC, such as providing energy, transmitting protumor signals, and evading inhibitory signals. In addition, exosomes induce angiogenesis by changing the biological characteristics of endothelial cells and directly regulating proangiogenic and propermeability factors. Furthermore, exosomes may lead to HCC metastatic invasion by epithelial-mesenchymal transformation, extracellular matrix degradation, and vascular leakage. Finally, we summarize the therapeutic usage of exosomes in the HCC microenvironment and attempt to provide a theoretical reference for modern antitumor agents designed to target these mechanisms.

## Background

The tumor microenvironment (TME) is the cellular environment in which the tumor develops. Apart from the tumor cells, the TME includes various cell types, extracellular matrix (ECM), growth factors, proteolytic enzymes and their inhibitors, and signaling molecules [1, 2]. TME influences tumor growth, metastasis, and ultimately prognosis. Therefore, the fundamental role of TME is to dynamically interact with malignant cells [3]. The TME contributes significantly to the pathogenesis of hepatocellular carcinoma (HCC). Indeed, by offering, inhibiting, or stimulating growth signals, this TME is an essential modulator of HCC development and progression and a source for identifying targets for potential therapeutic agents [4].

The interactions of HCC cells with the surrounding TME are based on complex systemic networks. In addition to direct cell-to-cell contact, intercellular communication through secreted factors plays a key role in intercellular signaling. Among these secreted factors, exosomes are the major components of extracellular vesicles (EVs), which range in size from 30 to 150 nm. EVs originate from multivesicular bodies (MVBs) and are generated by all cell types [5, 6]. Upon early to late endosome maturation, biomolecules are endocytosed and transported into early endosomes. In late endosomes, intraluminal vesicles (ILVs) are formed by inward budding of the endosomal membrane and result in a large MVB. MVBs can fuse with the plasma membrane, and the ILVs released into the extracellular space are referred to as “exosomes” [7, 8]. However, studies on the genesis and release of exosomes have revealed that apart from the sorting of cargo molecules, the procedure is tightly associated with energy mediators, such as SNAREs, Rabs, and Ras GTPases [9]. Exosomes are generated in the form of endocytosis, exocytosis, protein transport, and protein sorting. During this process, exosomes are packed with lipids, proteins, DNA, mRNA, miRNA, and other ncRNAs [5, 10], which are horizontally transferred from donor to recipient cells. Exosomes can carry biomolecules from tissues to body fluids [11–15]. These properties contribute to the role of exosomes in intercellular communication, i.e., shuttling of signaling molecules between nearby and remote cells [16–18]. The surface of the exosome contains a large number of molecules related to antigen presentation. In vivo and in vitro, exosomes have similar effects as antigen-presenting cells, which can induce and enhance immune responses. Exosomes have widely dissimilar sizes and contents and are heterogeneous in biological effects and targeting specificities. Thus, exosomes have attracted attention as important vehicles for specific signals in tumor progression, metastasis, immune modulation, angiogenesis, and tissue regeneration [19].

In the liver, exosomes are secreted by three main cell types: liver epithelia (i.e., hepatocytes and cholangiocytes), immune cells (i.e., T and B cells, dendritic cells, and NK cells), and nonparenchymal liver cells (e.g., liver stellate cells) [20–22]. Further evidence suggests a role for exosomes derived from different liver cells in the intracellular communication for the coordination of cell behaviors proper functioning. For example, exosomes derived from hepatocytes and cholangiocytes are important mediators of proliferation processes [20, 23]. T cell- and B cell-derived exosomes are involved in inflammation [24]. Exosomes derived from hepatic stellate cells (HSCs) may be involved in the pathogenesis of liver fibrosis [25]. Furthermore, primary hepatocyte-derived exosomes promote the activation of stellate cells, which in turn participate in liver disease progression [26]. Moreover, lipid-induced EVs derived from hepatocytes also cause an inflammatory macrophage phenotype [27]. Exosomes derived from the cells of other organs and tissues are involved in various types of liver disease [21]. For example, exosomes are involved in the progression of viral infections, including viral transmission, immune response, and antiviral effect [28, 29]; several studies have suggested that EVs increase with alcoholic hepatitis and show upregulation, even with excessive alcohol consumption [30, 31]. The role of exosomes in liver fibrosis by regulating connective tissue growth factor 2-dependent fibrogenesis in HSCs has also been reported [32].

**Fig. 1:**
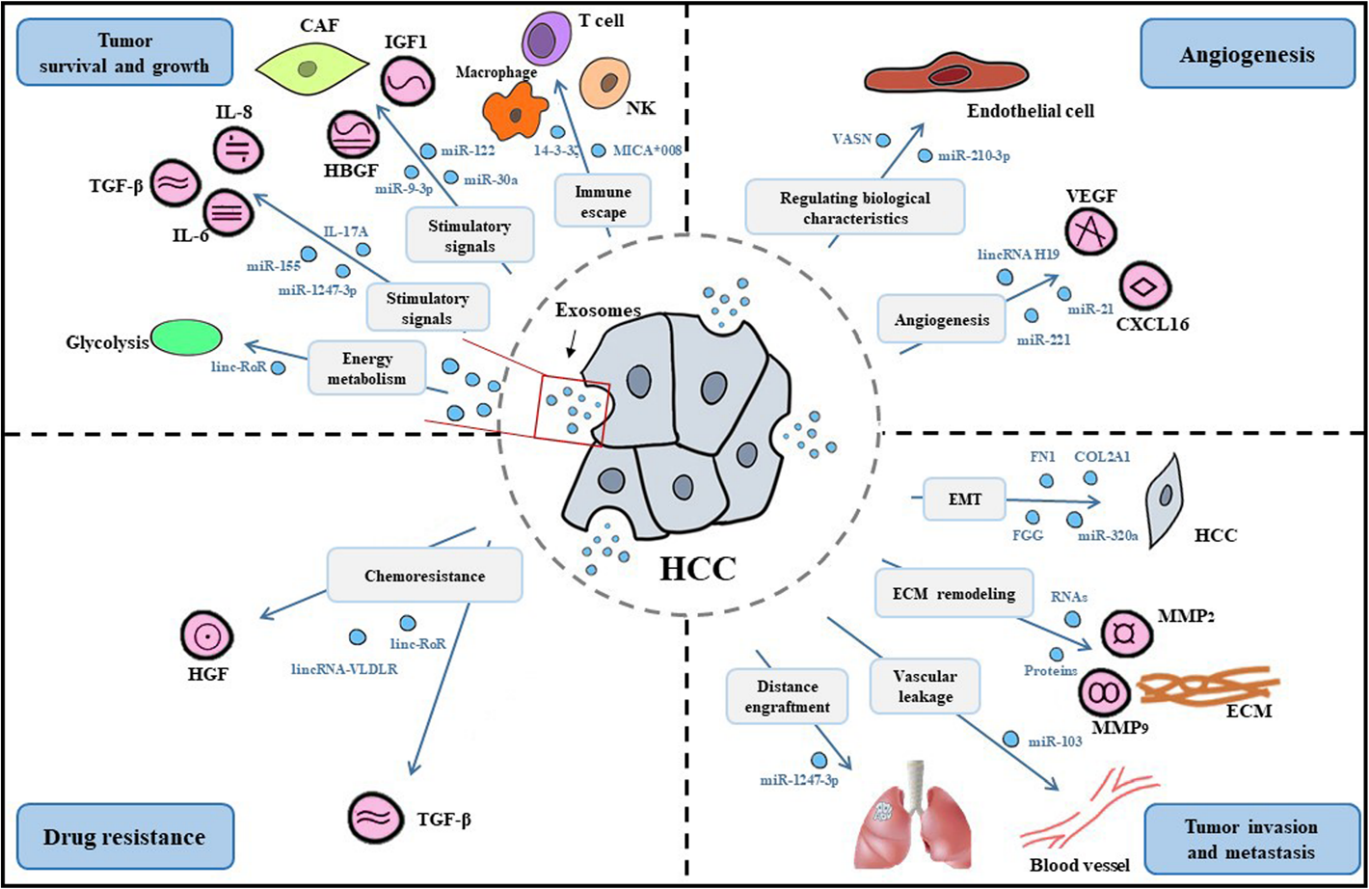
The functions of exosomes within TME in facilitating the development and progression of HCC. Exosome-mediated immune regulation is involved in modulation of the TME with immunosuppressive and tolerogenic characteristics. Multiple processes are involved in HCC, including tumor survival, growth, angiogenesis, invasion, and metastasis. The specific mechanisms involved in molding the TME for HCC include energy metabolism regulation, stimulatory signal supply, and inhibitory signal circumvention. In addition, exosomes induce angiogenesis by changing the biological characteristics of endothelial cells and directly regulating proangiogenic and propermeability factors. Additionally, exosomes may lead to HCC metastatic invasion by EMT, ECM degradation, and vascular leakage. Finally, the association between exosomes and drug resistance suggests the involvement of complex mechanisms of chemoresistance in the TME

However, what is the role of exosomes in HCC, and how is TME remolded by exosomes? With these considerations, we will focus here on the role of exosomes in setting up and modifying the HCC microenvironment that encourages tumor development and progression. We will discuss the specific mechanisms by which exosome-based communication regulates the immune response and benefits tumor survival, growth, angiogenesis, and metastasis (Fig. 1). We will also summarize the potential clinical applications of exosomes as therapeutic modalities.

## Role of the tumor microenvironment in promoting the development and progression of HCC

Despite advances in prevention, screening, and novel diagnostic and therapeutic technologies, HCC therapy has encountered a bottleneck. The 5-year survival rate of patients with HCC is still < 20% [33, 34], which indicates that HCC remains a highly lethal disease. An emerging concept is that the TME plays an important role in initiating and maintaining carcinogenesis [35]. The TME is a varying environment that describes the behavior of cancer. The TME is the cellular environment that tumor cells need for survival, growth, proliferation, and metastasis [36]. It is a complex and heterogeneous system that is orchestrated by two major groups of cellular and noncellular elements.

The cellular elements in the TME, such as HSCs, fibroblasts (cancer-associated fibroblasts or CAFs), immune cells (T lymphocytes, B lymphocytes, NK cells, natural killer T cells, and tumor-associated macrophages or TAMs), and endothelial cells (ECs), play a critical role in tumor-stromal interactions that can modulate biological activities of HCC. A study has shown that activated HSCs infiltrate the stroma of HCC and are associated with tumor proliferation [37]. One of the mechanisms is that HSCs reduce HCC central necrosis [38]. CAFs are the best studied stromal cells and are defined as loose fibroblasts found within the tumor mass. CAFs maintain the stem cell-like properties of HCC cells by secreting IL-6 [39] and promote tumor invasion and metastasis by secreting matrix metalloproteinase-9 (MMP-9) [40]. Immune cells are commonly described to restrain tumor development; however, when they intimately interact with transformed cells in the TME, these cells can alternatively propagate tumor progression [41, 42]. For example, the deficiencies and malfunction of immune cells can introduce an immunosuppressive microenvironment for tumor cells to achieve immune tolerance and evasion. ECs in TME are important suppliers of nutrients, oxygen, and various growth factors for HCC development.

The noncellular elements include growth factors, such as transforming growth factor-β (TGF-β), insulin-like growth factor (IGF), fibroblast growth factor (FGF), hepatocyte growth factor (HGF), and vascular endothelial growth factor (VEGF). The remaining noncellular elements also include proteolytic enzymes, ECM, and inflammatory cytokines. These factors can provide a pliable environment that encourages further HCC growth and propagation. TGF-β is an essential component of liver disease by participating in responses to surroundings and acting as a continual target for tumor-derived signals [43]. IGFs are mainly synthesized in the liver and are functionally related to HCC proliferation [44, 45]. Another important growth factor, FGF, is characterized by multiple functions that can mediate cell proliferation, angiogenesis, wound healing, and tissue repair [46]. HGF in the TME can promote malignant cell growth and survival [47]. Abnormal ECM in the TME can play a critical role in promoting angiogenesis and inflammation [48]. Many inflammatory cytokines, such as IL-6 and IL-8, as well as MMP-2 and MMP-9, participate in regulating the inflammatory microenvironment and contribute to the increased migratory and invasive potential of hepatocytes, thus facilitating cancer metastasis [49, 50].

Together, these data suggest that TME has a determinate protumorigenic effect and works as a booster in HCC progression and metastasis. TME has been considered to modulate the survival and growth of cell lines by providing inhibitory or stimulatory signals [4]. However, how are signals transmitted to and received from the TME? In recent years, growing evidence indicates that exosomes are important vehicles of specific signals in physiological scenarios [5]. In addition, exosomes have been increasingly studied as novel intercellular communication mediators for tumor onset and progression in TME [19].

## Exosome-mediated immune regulation in the HCC microenvironment

The immune microenvironment is shaped by intricate interactions between tumor cells and the host immune response. The evolution of cancer is strongly related to the tumor immune microenvironment. HCC shows a high degree of malignancy, and its poor overall survival outcome is due to the collapse of immune surveillance, which is closely associated with the suppression of host immune responses. Mounting evidence indicates that exosome-derived interactions are involved in the modulation of the microenvironment with immunosuppressive and tolerogenic characteristics. By regulating immune responses, tumors can evade immune destruction, and progression is ensured in this protumorigenic immune environment [51–54]. Pioneering work by Wang et al. [55] demonstrated that T cells could swallow HCC-derived exosomes containing 14-3-3ζ. The expression of 14-3-3ζ was upregulated in HCC cells and in tumor-infiltrating T lymphocytes (TILs). This process was related to impaired activation (CD69 expression), proliferation (Ki67 expression), and antitumor functions. In addition, 14-3-3ζ overexpression inhibits the vitality and proliferation of peripheral blood CD3+ T cells, promoting the differentiation of naive T cells from effector T cells to regulatory T cells. These findings suggested that HCC-derived exosomes containing 14-3-3ζ could inhibit the antitumor functions of TILs in the HCC microenvironment. NKG2D is a C-type lectin-like activating receptor expressed on all NK cells. Evidence has shown that NKG2D binding to its ligands mediates immune system activation and plays an important role in cancer immune surveillance [56, 57]. In fact, exosomes secreted by liver tumor cells induce NKG2D downregulation on NK cell surfaces, which leads to impaired cytotoxic function and is beneficial for HCC immune escape and progression [58]. Rao et al. demonstrated that HCC-derived exosomes can trigger DC-mediated immune responses by serving as vectors for a variety of antigens and remold the HCC tumor immune microenvironment [59]. Within the TME, tumor-derived exosomes play a key role in the polarization status of macrophages. In this setting, macrophages are observed to shift to a protumorigenic macrophage phenotype (M2 polarization), leading to tumor angiogenesis and metastasis [60, 61]. Melatonin is a hormone with certain cytotoxic and immunomodulatory effects, and this hormone can inhibit the functions of tumors. A recent study found that after treatment of hepatocellular carcinoma cells with melatonin, HCC-derived exosomes can alter the immunosuppressive status through the STAT3 pathway in macrophages [62].

HCC-derived exosomes can modulate the phenotype and function of immunocytes that can be beneficial for tumor cells to escape immune destruction. In turn, exosomes secreted by immunocytes in TME can affect antitumor immune responses. Mast cells can be stimulated by hepatitis C virus E2 envelope glycoprotein (HCV-E2) to secrete a large number of exosomes rich in miR-490. These exosomes are transferred into HCC cells and inhibit the metastasis of tumor cells via inhibiting the ERK1/2 pathway [63]. Exosomes derived from TAMs indisputably play a key role in immune suppression, angiogenesis, and tumor progression. For example, human macrophages can transfer exosomes with miR-142 and miR-223 to liver cells and inhibit the proliferation or growth of tumor cells [64]. In conclusion, HCC is characterized by the ability to employ different strategies to evade host immune surveillance, and exosomes are important promoters to alternatively propagate tumor progression by immune regulation.

## Exosomes mold the tumor microenvironment for HCC development and progression

### Tumor survival and growth: exosome-based communication in the HCC microenvironment functions as a determinant factor

Cancer development and progression relies on its complicated and heterogeneous microenvironment, which consists of a network of cellular and acellular constituents. This process is impacted by exosome-mediated transfer within the TME. The role and mechanisms of exosomes in the HCC microenvironment participating in multiple steps during tumor development and progression will be highlighted in this review (Table 1).

**Table 1:** The roles of exosomes within TME in HCC development and progression

Exosomal cargos	Regulation	Biological function	Mechanism	Reference
14-3-3ζ	Increased	Impair antitumor function	Inhibit the antitumor functions of TILs and the vitality and proliferation of peripheral blood CD3+ T cells	[55]
MICA*008	Increased	Impair cytotoxic function	Induce NKG2D downregulation on NK cell surface	[58]
HCC antigens	Increased	Promote immune responses	Activate immune response mediated by DCs	[59]
Not mentioned	Unvaried	Regulate immunosuppression	Alter the immunosuppressive status through STAT3 pathway in macrophages	[62]
miR-490	Increased	Inhibit metastasis	Mast cells are stimulated by HCV-E2 and secrete exosomes to inhibit the ERK1/2 pathway	[63]
miR-142, miR-223	Increased	Inhibit proliferation	Decrease reporter protein expression and endogenously express stathmin-1 and insulin-like growth factor-1 receptor	[64]
linc-RoR	Increased	Regulate energy metabolism	Activate microRNA-145/HIF-1α/PDK1 pathway and enhance the glycolysis process	[65]
		Induce chemoresistance	Activate TGF-β signaling and promote colony formation of CD133+ T-IC	[66]
miRNA	Increased	Promote migration and invasion	Induce TGF-β and TAK1 expression	[12]
miR-155	Increased	Promote formation and development	Promote inflammation and positively correlate with IL-6 or IL-8 levels	[67]
miR-1247-3p	Increased	Promote metastasis	Target B4GALT3 and activate β1-integrin–NF-κB pathway	[50]
miR-30a	Decreased	Promote proliferation and metastasis	Mediate Beclin 1 and Atg5-dependent autophagy	[68]
miR-320a	Decreased	Promote metastasis	Target PBX3 and MAPK pathway, induce EMT, and upregulate CDK2 and MMP-2 expression	[69]
miR-122	Decreased	Promote proliferation	IGF1 prevents intercellular exosomal transfer of miR-122	[70]
	Increase	Induce chemosensitivity	Induce chemosensitivity (5-FU and sorafenib)	[71]
miR-9-3p	Decreased	Promote proliferation	Regulate HBGF-5 and ERK1/2 expression	[72]
VASN	Increased	Promote HUVECs cells migration	Not mentioned	[73]
RNAs, miRNAs	Polytropic	Associated with the degree of lumen formation	Not mentioned	[74]
miR-210-3p	Increased	Increase angiogenesis	Inhibit the expression of SMAD4 and STAT6 in ECs	[75]
lincRNA H19	Increased	Increase angiogenesis	Increase VEGF release and the production of VEGF-R1	[76]
miR-221	Increased	Increase angiogenesis	Activate SAND/NF-κB pathway and upregulate CXCL16 expression	[77, 78]
miR-21	Increased	Increase angiogenesis	Activate the STAT3/VEGF pathway	[77, 79]
Not mentioned	Not mentioned	Induce chemoresistance	Activate HGF/c-Met/Akt pathways and restrain apoptosis	[80]
Fibronectin1 COL2A1, FGG	Increased	Promote metastasis	Induce either partial or total EMT	[81]
RNAs, proteins	Polytropic	Promote migration and invasion	Activate PI3K/AKT and MAPK pathways in MIHA and increase MMP-2 and MMP-9 secretion	[82]
miR-103	Increased	Promote metastasis	Inhibit the expression of VE-Cad, p120, and ZO-1	[83]
AFP	Increased	Promote immune responses; Inhibit proliferation	Enhance CD8+ T lymphocytes response, improve IFN-γ and IL-2 expression	[84]
HSP	Increased	Promote immune responses	Improve tumor immunogenicity and induce NK cell responses	[85]
PD-L1	Increased	Promote proliferation	Suppress T cell activation in the draining lymph node	[86]
miR-26a	Increased	Inhibit proliferation	Bind to HepG2 cells via the scavenger receptor class B type 1-Apo-A1 complex	[87]
Doxorubicin	Increased	Inhibit proliferation	imDCs were engineered to express Lamp2b fused with v integrin-specific iRGD peptide	[88]
lincRNA-VLDLR	Increased	Induce chemoresistance	Increase ABCG2 expression and restrain apoptosis	[89]

#### Regulating energy metabolism

Tumor cells produce hypoxia-inducible factor (HIF) as an important mediator of tumor metabolism, which regulates the expression of target genes, such as erythropoietin, iron metabolism-related genes, VEGF, GLUT1, glycolytic enzymes, heme oxygenase HO2-1, and inducible nitric oxide synthase. Thus, tumor cells can reprogram energy metabolism under hypoxic conditions and achieve malignant proliferation [90]. Takahashi et al. [65] found that under hypoxic conditions, hepatocellular carcinoma cells promote the secretion of linc-ROR-containing exosomes and escalate the expression of miRNA-145, HIF-1α, and its downstream regulatory protein pyruvate dehydrogenase kinase isozyme 1 (PDK1). Furthermore, knockdown of linc-RoR has a noteworthy effect on the recovery of the expression of these three proteins. PDK1 is a hypoxia stress protein that regulates mitochondrial function and enhances glycolysis by reducing the entry of pyruvate into the tricarboxylic acid cycle. Therefore, HCC-derived exosomes can activate the microRNA-145/HIF-1α/PDK1 pathway, which may enhance the glycolysis process and resist a hypoxic environment. However, further investigation is required to determine the specific mechanisms.

#### Providing stimulatory signals

Exosomes secreted by HCC regulate the surrounding microenvironment and create conditions conducive to tumor growth and development. The multifarious characteristics of growth factors in HCC have been well studied by many researchers. The development of HCC is associated with TGF-β overexpression [91]. Furthermore, TGF-β overexpression in HCC patients gives significant advantages to tumor growth by facilitating the advancement of a favorable microenvironment [92]. Takayuki et al. demonstrated that exosomes packed with miRNA secreted by HCC cells promote tumor motility, invasion, and dissemination by inducing the expression of TGF-β and TGF-β-activated kinase-1 [12]. An inflammatory environment effectively facilitates HCC formation and development. In this setting, exosomes have been shown to play an important role in mediating the regulation of the inflammatory microenvironment to promote cancer progression and metastasis. For instance, when exposed to arsenite, HCC cells produce miR-155-rich exosomes that enhance inflammation and positively correlate with IL-6 or IL-8 levels [67]. Another example involves the bidirectional communication between HCC and its exosome-mediated inflammatory microenvironment, that is, while the inflammatory microenvironment promotes tumorigenesis, the tumor also creates an inflammatory environment that promotes its own development. High-metastatic HCC cells secrete exosomes with miR-1247-3p, activating β1-integrin–NF-κB signaling in fibroblasts by directly targeting B4GALT3. In fact, activated CAFs further promote pleiotropic actions, such as secreting proinflammatory cytokines (IL-6 and IL-8) that govern the progression of cancer [50].

Conversely, exosomes secreted from surrounding nontumor cells can transmit growth signals, promoting tumor expansion and aggressiveness. When liver injury occurs, hepatic exosomes activate HSCs through increasing IL-17A production [93]. IL-17A is associated with the progression of HCC [94]. In vitro and in vivo studies have demonstrated that HSC-conditioned media prominently promoted HCC cell proliferation and development: activated HSCs infiltrate the stroma of HCC and are involved in the proliferation of HCC cells [37]; in line with this finding, co-cultured HSCs and HCC cells can promote tumor growth and invasiveness in nude mice [38]. These findings indicate that signals can be transmitted to HSCs via exosomes, potentially promoting the onset and progression of HCC. However, the specific mechanisms by which exosomes trigger healthy stromal cells to promote the malignant behavior of cancer cells require further investigation.

#### Eluding inhibitory signals

In the past decade, there has been increasing emphasis on the potential role of exosome-mediated HCC to evade inhibitory signals, thereby promoting the malignant behavior of cancer cells. miR-30a, as a cancer suppressor, has been demonstrated to inhibit migration and invasion in HCC [95]. Exosomes packed with miR-30a derived from HSCs and LX-2, which were activated by TGF-β1, could notably be downregulated [96]. A recent study showed that HCC promotes vascular invasion, metastatic potential, and recurrent disease by downregulating miR-30a in tissues and cell lines [68]. In addition, CAF-derived exosomes overexpressing miR-320a can inhibit tumorigenesis, which implies that a possible cause of HCC progression mediated by CAFs is linked with a deficiency in antitumor miR-320a in CAF-derived exosomes [69]. miR-122 has liver-specific antiproliferative characteristics [97]. Basu et al. detected the intercellular transfer of miR-122 via exosomes, but exosomal transfer was prevented by IGF1 in HCC, suggesting that the expression of exosomal miR-122 is prevented to ensure tumor proliferation [70]. Similarly, HCC can reduce the expression level of serum exosomes with miR-9-3p to promote tumor cell viability and proliferation [72]. Most studies on the relevance of miRNA in cancer biology have been performed in vitro, which has some limitations. However, numerous studies suggest that exosome-derived miRNAs can mediate the biological information transmission between HCC and TME and participate in the biological process of HCC in multiple forms.

### Exosome-induced angiogenesis: the tumor microenvironment has a complex impact

Active angiogenesis has been suggested as the leading cause of rapid tumor growth, early metastasis, and poor survival [98]. In addition, exosomes regulate intercellular communication through proteins, nucleic acids, and other cargo to alter the TME, inducing the formation of vascular lumens and ultimately promoting the malignant proliferation and invasive phenotype of tumor cells.

#### Regulating biological characteristics of endothelial cells

ECs are recognized as essential components of TME by providing a conduit to nutrients, oxygen, and various growth factors in the course of tumor development. Several proteins, such as VEGF and angiopoietin, have been implicated in regulating the biological and physical characteristics of ECs. Additionally, tumor cell-derived exosomes containing functional proteins and miRNAs can affect the biological fate of endothelial cells. For example, Huang et al. confirmed that HepG2-derived vasorin (VASN, a type I transmembrane protein) can be effectively delivered into human umbilical vein endothelial cells (HUVECs) via receptor-dependent endocytosis of exosomes, which in turn induced migration of HUVECs [73]. In addition, the number of HepG2 exosomes was firmly associated with the degree of lumen formation [74]. Exosome-mediated miRNA transfer also modulates the biology of ECs. Lin et al. revealed that HCC cells secrete exosomes containing miR-210-3p (miR-210), which can promote the tube formation of ECs in vitro. Specifically, exosomal miR-210 can enhance angiogenesis by inhibiting the expression of SMAD4 and STAT6 in ECs [75].

#### Promoting tumor angiogenesis

In addition to EC tubulogenesis-related angiogenesis, exosomes are involved in other mechanisms promoting tumor angiogenesis. VEGF, as the most powerful proangiogenic cytokine, has the ability to transduce signals necessary for the formation of the three-dimensional vascular tube and in regulating vascular permeability. Conigliaro et al. found that CD90+-derived exosomes expressed high levels of lncRNA H19, which could substantially increase VEGF release and the production of VEGF-R1, hence stimulating angiogenesis. After lncRNA H19 silencing, the expression level of VEGF protein decreased significantly [76]. Therefore, exosomes could regulate the transcription of target cell-related genes and promote angiogenesis. In addition, exosomes are involved in the regulation of the HCC angiogenesis-related signaling pathway. The expression of miR-221 and miR-21 are elevated in HCC and HCC-derived exosomes [77]. miR-221 can stimulate the activation of the SAND/NF-κB pathway and upregulate angiogenic factor CXCL16 expression, which plays a role in promoting angiogenesis [78]. miR-21 can activate the STAT3/VEGF signaling pathway and shape a vascular microenvironment for HCC [79]. As a response to this vascular microenvironment, HCC shows accelerated growth kinetics. These observations suggest the role of HCC-secreted exosomes in remodeling TME and promoting tumor angiogenesis.

### Exosome-mediated HCC invasion and metastasis: local and distant microenvironments act as critical sanctuaries

Metastasis is an important feature distinguishing tumors from non-neoplastic diseases. Before the occurrence of distant metastasis, tumor-secreted exosomes can activate invasive-related soluble factors to remold the local and distant TME for the implementation of tumor metastatic invasion.

#### Exosomes and EMT

Epithelial-mesenchymal transformation (EMT) is a reversible dedifferentiation process characterized by the loss of epithelial characteristics and the acquisition of typical mesenchymal properties, which is considered the central mechanism of HCC invasion and metastasis [99, 100]. A recent study indicated that HCC cell-derived exosomes can increase HGF levels in the cell culture supernatant [80]. HGF has been reported to be the most potent growth factor for hepatocytes and its receptor. In the TME, HGF has been suggested to cause HCC phenotypical changes through EMT, migration, and invasion [101, 102]. Although not directly verified, there may be a correlation between HCC-derived exosomes and the induction of EMT. A recent study confirmed this correlation from the opposite perspective. The differential expression of exosome proteins from conditioned media of HCC cells was analyzed by Karaosmanoğlu and colleagues. These authors found that some specific proteins were highly enriched in exosomes secreted by slug-overexpressing HCC cells, which were involved in the induction of either partial or total EMT [81]. In addition, exosomes derived from CAFs could transfer miR-320a to HCC cells, which could suppress EMT and videlicet, while the loss of antitumor miR-320a in the exosomes of CAFs could induce EMT and promote tumor progression [69]. However, the role of exosomes in EMT initiation requires further elucidation.

#### Exosome-mediated ECM remodeling promotes local tumor invasion

Some major components of ECM include collagens, laminins, fibronectin, glycosaminoglycans, and proteoglycans, which play a very crucial role in changing the phenotypic and functional characteristics of HCC and stroma cells [103]. Exosomes can set up a mechanically stiff microenvironment by enhancing the reorganization of ECM, which ultimately promotes tumor metastasis. He et al. found that exosomes derived from highly metastatic HCC cell lines were packed with proteins such as met, caveolin, and S100. After ingestion by the normal liver cell line MIHC, these exosomes contribute to enhancing the migratory and invasive potential of MIHC. In addition, the expression of Akt, phosphorylated MEK1/2, and metalloproteinases (MMP-2 and MMP-9) was increased significantly [82]. Based on these data, HCC-derived exosomes trigger the production of MMPs and enable the degradation of ECM to energize local invasion and cell migration.

#### Exosomes promote vascular leakage and benefit the engraftment of tumor cells

Exosomes derived from tumors have attracted more attention for the conversion of vascular permeability, which ultimately leads to tumor metastatic dissemination. Cell-to-cell junctions between endothelial cells are dependent on VE-cadherin, catenin (p120-, p0071-, and β-catenin), ZO-1, and claudin-5 [104]. Recent evidence indicates that miR-103 derived from hepatoma cells could be transmitted to endothelial cells via exosomes, which then inhibit the expression of VE-cadherin, p120-catenin (p120), and ZO-1 to attenuate the integrity of endothelial junctions, induce vascular leakiness, and consequently facilitate tumor metastasis [83]. Furthermore, clinical data show that high serum exosomal miR-1247-3p levels are associated with lung metastasis in HCC patients. The specific mechanism by which exosomes facilitate tumor cell arrival to the lungs and achieve tumor engraftment is the conversion of fibroblasts. High-metastatic cells secrete exosomal miR-1247-3p to convert fibroblasts to CAFs in the lung premetastatic niche by targeting B4GALT3 [50]. Taken together, these data suggest that the intrinsic interactions of tumor-derived exosomes with target cells in a tumor environment further illuminate new mechanisms of HCC expansion and metastasis.

## The therapeutic usage of exosomes in the HCC microenvironment

As exosome-based communication in the HCC microenvironment can function as a determinant factor to promote the malignant behavior of cancer cells, exosomes in the HCC microenvironment may provide an opportunity for targeted therapeutic intervention to eventually prevent HCC and prolong the duration of survival for HCC patients. Because exosome-mediated immune regulation in TME plays an important role in the occurrence and development of HCC, it is reasonable to expect that the exosome-based communication system is beneficial to stimulate the immune response and to mediate HCC immunotherapy. In addition, increasing interest is focused on the utility of exosomes as therapeutic tools for drug and biological molecule transfer.

### Exosome-based immunotherapy in the HCC microenvironment

Enhancing immunity is the basic therapeutic approach to HCC therapy [105]. Exosomes have been shown to enhance tumor immunogenicity and antitumor immune responses. Rao et al. revealed that tumor cell-derived exosomes expressed an array of HCC antigens, which significantly activated the immune response mediated by dendritic cells (DCs) in a preclinical model, ultimately reshaping the HCC immune microenvironment and improving antitumor effects [59]. Moreover, DC-derived exosomes have been demonstrated to be useful as cancer vaccines for immunotherapy. A study using exosomes derived from α-fetoprotein (AFP)-expressing DCs (DEXAFP) to explore the relationship between the tumor immune microenvironment and HCC suppression is ongoing. Benefitting from the enhanced CD8+ T lymphocyte response, the expression levels of IFN-γ and IL-2 were improved, while CD25+Foxp3+ regulatory T (Treg) cells, IL-10, and TGF-β levels were decreased in tumor sites. DC-derived exosomes can significantly impede HCC growth and prolong survival rates in mice [84]. In terms of exosome-mediated immunotherapy, another example showed that exosomes derived from HCC cells were enriched for stress-induced heat shock protein (HSP) to improve tumor immunogenicity and induce NK cell responses through the upregulation of inhibitory receptor CD94 and downregulation of activating receptors (CD69, NKG2D, and NKp44) [85]. Immune checkpoint protein inhibitors have revolutionized cancer treatment; however, anti-PD-L1/PD-1 therapy is only effective in 10–30% of patients [106]. A recent study showed that exosomes derived from tumors with PD-L1 could suppress T cell activation in the draining lymph node, suggesting that tumor growth could be suppressed by exosomal PD-L1 blockade. Inhibiting the release of PD-L1 exosomes might overcome resistance to current antibody approaches [86].

### Exosomes as therapeutic tools in the HCC microenvironment

Accumulating evidence has shown that exosomes could present promising drugs and biological molecule delivery vehicles for cancer therapy due to biocompatibility and stability, endure modification, and cross the blood-brain barrier [107, 108]. Mesenchymal stem cells (MSCs) produce massive exosomes. When adipose tissue-derived MSC (AMSC) exosomes were transfected with miR-122 expression plasmids, miR-122 can be effectively packaged into secreted exosomes and transferred to HCC cells, thereby rendering HCC cells sensitive to chemotherapy agents (5-FU and sorafenib) [71]. Another study provided a novel approach to package the contents into exosomes. Functional miR-26a was loaded via electroporation into the exosomes of engineered 293 T cells, and then, these engineered exosomes could bind selectively to HCC cells through the scavenger receptor to reduce the rates of cell migration and proliferation [87]. In an example of chemotherapeutic delivery, exosomes were used as targeted drug delivery vehicles for low immunogenicity and toxicity. Immature dendritic cells (imDCs) were engineered to express a fully characterized exosome membrane protein (Lamp2b) fused with v integrin-specific iRGD peptide for the targeted transportation of exosomes loaded with doxorubicin to tumor tissues [88]. Such targeted exosomes were injected intravenously, which specifically delivered doxorubicin to the tumor tissue, resulting in the inhibition of tumor growth without significant toxicity.

### The relationship between chemosensitivity and exosomes in the HCC microenvironment

HCC has been indicated as resistant to traditional chemotherapeutic strategies. Although many unanswered questions remain, the association between exosomes and drug resistance has provided important insights into a promising new strategy for HCC chemoresistance. Studies have found that CD133+ T-IC [109] and the ATP-binding cassette family [110] are important factors leading to HCC drug resistance. Recent studies have shown that exosomes have been implicated in promoting resistance to chemotherapy by promoting the expression of CD133+ T-IC and ABCG2 in the TME. Takahashi and colleagues demonstrated that exosomal linc-ROR from HCC can be significantly upregulated after the incubation of tumor cells with diverse anticancer agents, thereby activating TGF-β signaling and promoting colony formation of CD133+ T-IC, resulting in the reduction of cancer cells sensitive to chemotherapy [66]. In addition to the regulation of CD133+ T-IC by exosomal linc-ROR, another mechanism associated with HCC chemoresistance is the upregulation of ABCG2 by exosomal lincRNA-VLDLR. Based on these observations, RNAi-mediated knockdown of linc-VLDLR can reduce cell viability, arrest the cell cycle in the G1/S phase, and promote apoptosis [89]. Another in vitro study revealed that HGF/c-Met/Akt signaling pathways can be triggered by HCC cell-derived exosomes, which play an important role in restricting sorafenib-induced apoptosis and contribute to drug resistance of liver cancer [80]. These examples of exosome manipulation reveal complex mechanisms of chemoresistance in the TME and may offer potential therapeutic strategies for HCC.

## Conclusions

TME is the cellular environment that malignant cells need for survival, growth, proliferation, and metastasis. Exosomes, as a novel intercellular communication mediator in the TME, have attracted attention for promoting tumor onset and progression. The present review summarized the role of exosomes in setting up and modifying the TME for the development and progression of HCC. Here, we revealed the specific mechanisms through which exosome-based communication within the HCC microenvironment promoted the malignant behavior of cancer cells. It is possible that exosome-mediated therapy in HCC patients could involve the multifaceted roles of exosomes in the TME. Future work is needed to illuminate the potential mechanisms by which exosomes have effector functions in the TME to provide an opportunity for targeted therapeutic intervention based on exosome communication systems.

## Data Availability

The material supporting the conclusions of this review is included within the article.

## Abbreviations

CAFs: Cancer-associated fibroblasts
DCs: Dendritic cells
ECM: Extracellular matrix
ECs: Endothelial cells
EMT: Epithelial-mesenchymal transformation
EVs: Extracellular vesicles
FGF: Fibroblast growth factor
HCC: Hepatocellular carcinoma
HGF: Hepatocyte growth factor
HIF: Hypoxia-inducible factor
HSCs: Hepatic stellate cells
HSP: Heat shock protein
HUVECs: Human umbilical vein endothelial cells
IGF: Insulin-like growth factor
ILVs: Intraluminal vesicles
MMPs: Matrix metalloproteinases
MSCs: Mesenchymal stem cells
MVBs: Multivesicular bodies
PDK1: Pyruvate dehydrogenase kinase isozyme 1
TAMs: Tumor-associated macrophages
TGF-β: Transforming growth factor-β
TILs: Tumor-infiltrating T lymphocytes
TME: Tumor microenvironment
VASN: Vasorin
VEGF: Vascular endothelial growth factor
